# The Effect of Gravity on Flame Spread over PMMA Cylinders

**DOI:** 10.1038/s41598-017-18398-4

**Published:** 2018-01-09

**Authors:** Shmuel Link, Xinyan Huang, Carlos Fernandez-Pello, Sandra Olson, Paul Ferkul

**Affiliations:** 1 University of California Berkeley Department of Mechanical Engineering, Berkeley, CA USA; 2 NASA Glenn Research Center at Lewis Field, Cleveland, OH USA

## Abstract

Fire safety is a concern in space travel, particularly with the current plans of increasing the length of the manned space missions, and of using spacecraft atmospheres different than in Earth, such as microgravity, low-velocity gas flow, low pressure and elevated oxygen concentration. In this work, the spread of flame over a thermoplastic polymer, polymethyl methacrylate (PMMA), was conducted in the International Space Station and on Earth. The tests consisted of determining the opposed flame spread rate over PMMA cylinders under low-flow velocities ranging from 0.4 to 8 cm/s and oxygen concentrations from 15% to 21%. The data show that as the opposed flow velocity is increased, the flame spread rate first increases, and then decreases, different from that on Earth. The unique data are significant because they have only been predicted theoretically but not been observed experimentally before. Results also show that flame spread in microgravity could be faster and sustained at lower oxygen concentration (17%) than in normal gravity (18%). These findings suggest that under certain environmental conditions there could be a higher fire risk and a more difficult fire suppression in microgravity than on Earth, which would have significant implications for spacecraft fire safety.

## Introduction

Fire safety in reduced gravity is an important concern for space travel^1–3^. Particularly, the fire risk will increase as the time spent in space is increased with the operation of proposed space missions, such as the proposed Exploration Gateway Platform^4^. There is an even higher fire risk of using Space Exploration Atmospheres (SEA) with lower pressure and higher oxygen concentrations than on Earth. The flammability of solid materials is a measure of their fire hazard, and consequently is often used to characterize the fire risk of a material. Flammability of material is typically characterized by its ignitability, flame spread behavior, heat release rate, and toxicity. The most effective fire safety strategy is to prevent ignition. However, once ignited, the spread of flame presents a significant safety risk to the space travel. Thus, experiments on flame spread are often used to determine the fire hazard of a material and the corresponding fire-extinguishing strategy^5^.

**Figure 1:**
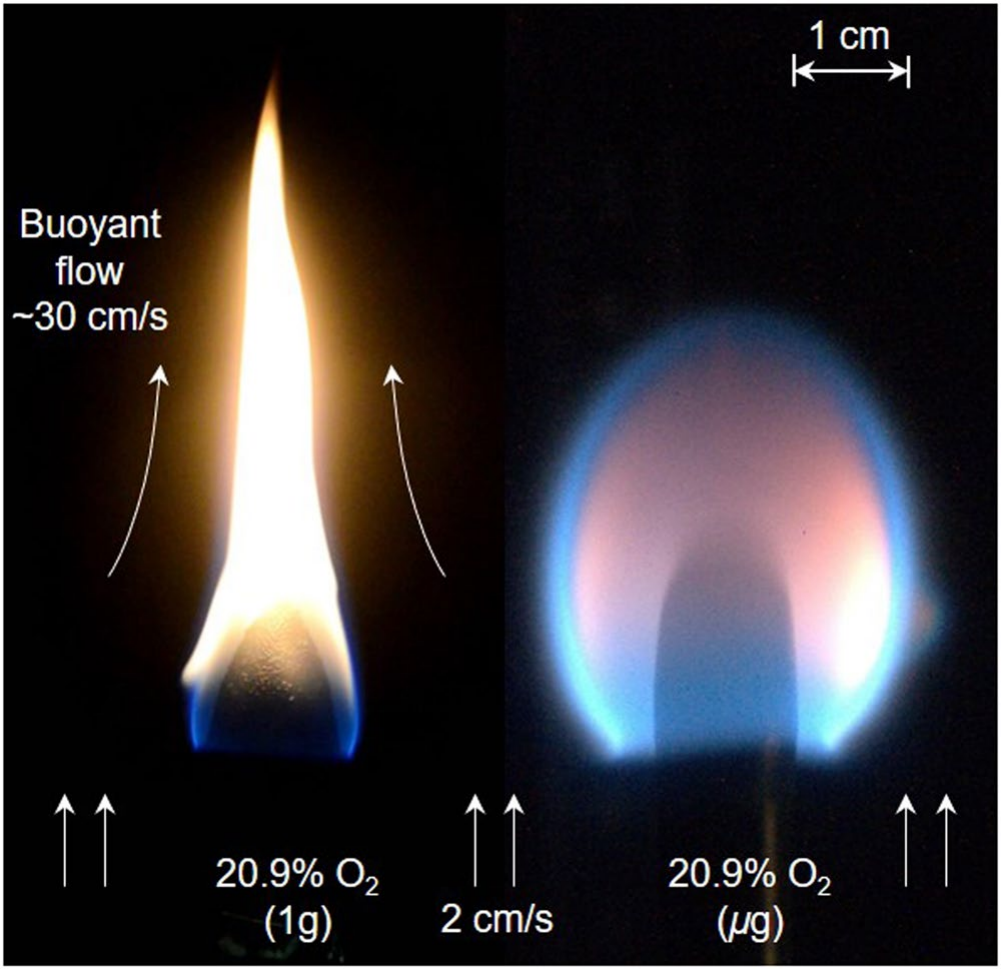
Photo of the flame spread normal gravity and microgravity at ${X}_{{{\rm{O}}}_{2}}$ = 20.9%, where all samples are cast black PMMA rod.

The spread of flame over the surface of a solid combustible material is a complex process involving the interaction between the solid phase (heat transfer, thermal decomposition, gasification) and the gas phase (transport, mixing, chemical kinetics)^6,7^. Flame spread is very much affected by external environmental conditions which may be very different in a spacecraft from on Earth, e.g. microgravity, low-velocity flow, variable oxygen concentrations, and reduced pressures^8,9^. A clear example is that of Fig. 1 which shows a comparison of the flame characteristics between normal gravity and microgravity for a flame spreading over the surface of a PMMA rod under the same ambient pressure and oxygen concentration. In normal gravity (Fig. 1, left) buoyancy induces an upward gas flow (~30 cm/s) that contracts and enlarges the flame, and enhances the burning of the pyrolyzed fuel. In microgravity (Fig. 1, right) at a gas flow of 2 cm/s, significantly smaller than the buoyant flow in normal gravity, the flame is rounder due to radial mass diffusion and bluer due to a lower soot concentration.

In spacecraft, gravity is reduced (often to microgravity) and there are low-velocity flows are of the order of 0.1 m/s, due to the heating, ventilation and air conditioning (HVAC) system^1^. Such flows are significantly smaller than those induced by the flame-induced buoyant flow on Earth^10^. Also, a reduced pressure is desirable to reduce preparation time for human’s extravehicular activities, although it requires elevated oxygen concentration to keep the spacecraft in Normoxic conditions (constant oxygen partial pressure)^11^. Currently, there is not sufficient knowledge regarding the fire-spread behavior of materials in environments similar to those expected in future spacecraft. Nor there is a testing methodology specifically developed to determine the fire hazards of materials under those environments.

There have been limited studies on the microgravity combustion of solid fuels, primarily because of the difficulty of conducting those studies^12–20^. Those studies indicate that under reduced gravity and low flow velocity the ignition delay becomes shorter, flame structure becomes rounder with less intense luminance, and flame spread rate may be reduced. Very few experimental studies have reported the flame spread in SEA spacecraft environments, particularly for relatively thick material. This is because the extensive time required to conduct the experiments is only possible in long-term microgravity facilities like the International Space Station (ISS).

In this work, experiments on the spread of flames in an oxidizing gas flow opposing the direction of flame spread are reported both in microgravity (μg) and normal gravity (1 g). The microgravity experiments were conducted in ISS as a part of the Burning and Suppression of Solids-II (BASS-II) project^21–23^. The scientific objective of the BASS-II opposed flow flame spread tests was to understand further the mechanisms controlling the spread of flames over the surface of solid combustible materials, particularly in a microgravity environment. For this purpose, the spread of flames over polymethyl methacrylate (PMMA) rods of different diameters were tested subjected to low opposed flow velocities and oxygen concentrations lower than air. PMMA is a thermoplastic used extensively on Earth but also potentially used for some specific components (e.g. windows) in planned spacecraft. The reduced oxygen concentration allows for the determining of the limiting oxygen concentration (LOC) for flame spreading in a candle-like flame. LOC is a parameter used in fire safety to determine the relative flammability of materials^5,24^. Also, comparing the ISS-based data to ground-based data provides further information on the mechanisms of flame spread in a spacecraft environment.

## Experiments

**Figure 2:**
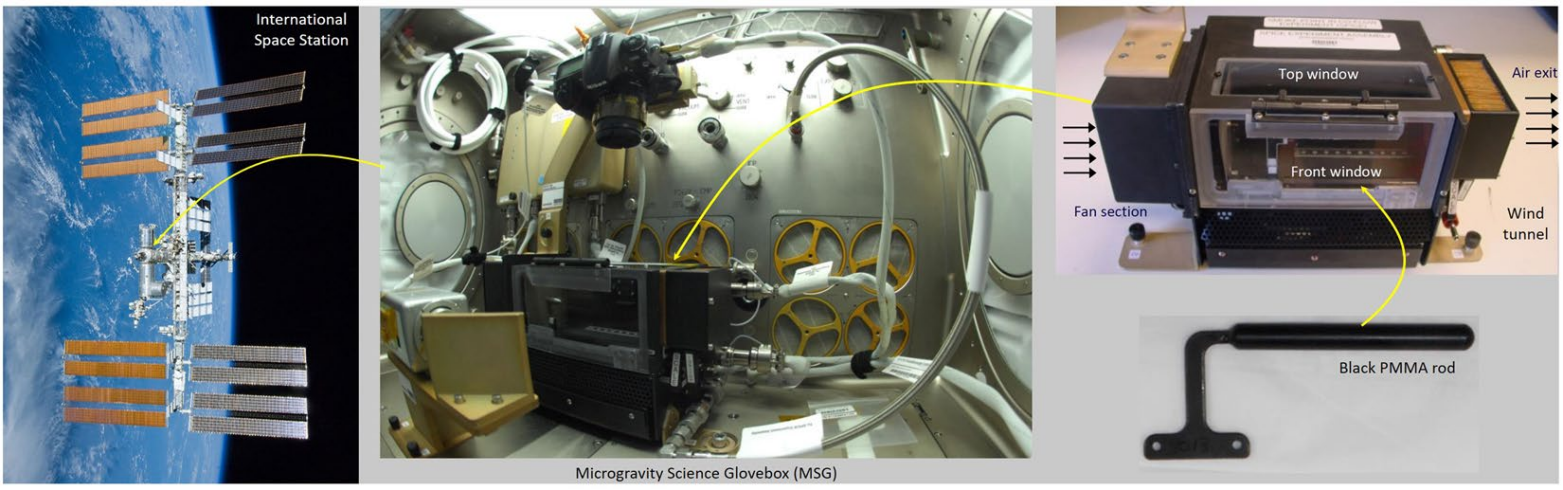
Microgravity Science Glovebox (MSG) Facility working volume aboard the International Space Station (ISS), with the Suppression of Solids-II (BASS-II) experiment hardware and black PMMA rod. Images courtesy of NASA https://www.nasa.gov/mission_pages/station/research/news/bassII.

Microgravity flame spread tests were conducted in the BASS-II hardware placed inside the Microgravity Science Glovebox (MSG) in the ISS Destiny Lab. Photographs of the experiment are shown in Fig. 2. The BASS-II hardware provides a contained atmosphere in which it is possible to conduct fire safety experiments^21–23^. It consists of a flow duct, still camera, video camera, external control box, and associated plumbing and mounting systems. The black anodized 76 mm by 76 mm rectangular flow duct was originally built to perform gas jet diffusion flame studies and was modified to accommodate solid samples for the BASS-II experiments.

The flow was generated using a small fan at the upstream end of the duct. The voltage to the fan was varied to change the flow velocity through the duct. Also, optional flow restrictors were used at the fan inlet to increase the pressure drop and reduce the flow through the duct, thus providing a low-velocity flow. The flow then passed through a honeycomb flow straightener and an inlet screen to reduce swirl. An omnidirectional spherical hot wire anemometer was positioned between the honeycomb and the screen to measure the steady-state flow velocity through the duct.

The test section was 17 cm long. Inside the test section was a nozzle for nitrogen flow, a moveable scale, and an Oriel 71768 thermopile detector with a CaFl windows with a spectral range of 0.13 to 11 μm in the downstream top back corner of the duct. The test section of the duct had two orthogonal windows, and the top one provided a mounting rail system for the cylindrical samples. The top window was used by a Nikon D300 12.3 mega-pixel digital color still camera with a 60-mm lens that provided 4320 × 2968-pixel images, specifically, providing a resolution of 34 pixels/mm in sample. The duct exit contained a perforated copper plate followed by a brass screen to provide heat rejection and a cold surface for soot deposition. The front window was opened to provide access to the test section for sample and igniter change-out. The front window also had interlocks for the igniter and nitrogen flow. A Panasonic color video camera WV-CP654 (760 × 480 pixel) with a turning mirror looked in the front window. The video camera had a built-in digital overlay that displayed the nitrogen flow rate, fan voltage, hot wire anemometer reading, and the radiometer signal.

The fan voltage was calibrated with the hot wire reading at the end of every ops day. The radiometer signal was not calibrated but provided a measure of the flame dynamics and steadiness. The external control box had controls for the fan voltage level, nitrogen flow level, and enable switches for the igniter and nitrogen, and a radiometer gain level setting. The samples were manually ignited with a Kanthal A-1 29 gauge hot-wire igniter with a nominal hot wire resistance of 1.1 ohms, powered by 5 V when the astronaut pulled the deployment lever to move the igniter into position. Samples were burned within the duct, and the combustion products exit the duct and mix with the gas in the work volume.

**Figure 3:**
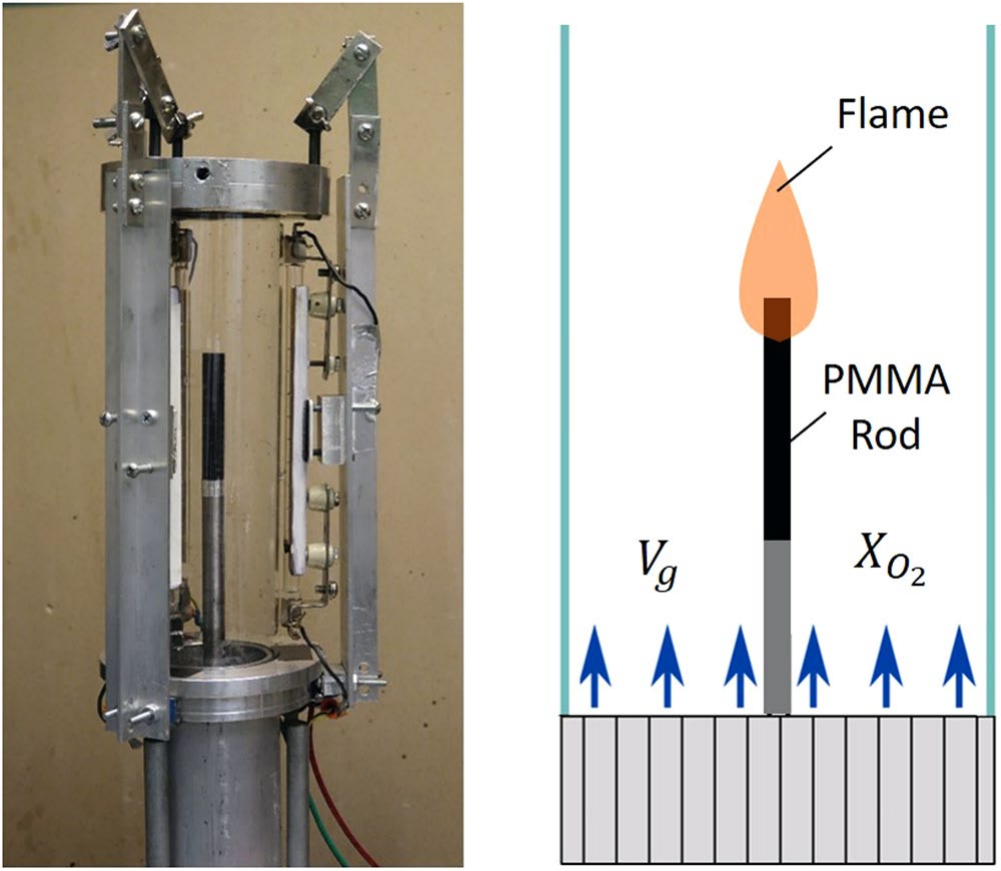
Normal gravity experimental setup and schematic diagram.

The normal gravity experimental apparatus had the same basic characteristics of the microgravity one and similar to ASTM limiting oxygen test^25,26^. It consisted of a small-scale cylindrical flow duct where the cylindrical fuel sample was placed, and supporting instrumentation (Fig. 3). The vertically oriented flow duct had an outer diameter of 75 mm, an inner diameter of 70 mm, and a length of 254 mm. Upstream from the flow duct, there was a flow mixing chamber, with inlets for both pressurized air, or oxygen, and nitrogen. These pressurized gases were metered via sonic orifices that allowed for controlled flows of gas with a prescribed oxygen (balance nitrogen) concentration between 16 and 21% at velocities from 0 (no flow) to 350 cm/s. Between the flow mixing chamber and the flow duct was a flow homogenizer section with approximately 50 mm of borosilicate glass beads. The flow duct provided for a consistent and uniform flow of the oxidizer gas to the test section where the fuel sample was located.

The microgravity tests were conducted with rods of cast black PMMA with diameters of 6.4, 9.5, and 12.7 mm and were 57.2 mm in length. The black PMMA was selected, instead of clear PMMA, to avoid the influence of in-depth radiation. All rods tested had rounded (hemispherical) ends to minimize the flow disturbance associated with the abrupt bluff body transition at the sample’s downstream edge. Oxygen concentration, defined by the volume fraction, was varied between 16% and 21%, and flow velocities were varied between 0.4 and 8 cm/s. Keeping the flow oxygen concentration constant, each PMMA sample was ignited at a relatively high opposed flow velocity by a hot coil, and the steady-state flame spread was achieved within 1 min. Then, the opposed flow velocity was subsequently reduced until extinction was observed, pausing (about 30 s) along the way to allow for steady state flame spread at each new flow velocity.

For the normal gravity tests, besides samples of the same three diameters tested in microgravity, additional 3.2-cm and 1.9-cm thick samples were also tested. Each cylindrical fuel sample was supported from below with a steel rod holder which had the same diameter as the sample and was placed in the axis of the flow duct. Then, the oxidizer could flow smoothly over the sample’s surface. The ignition was achieved by using a small methane diffusion flame under a still air. When the flame became robust, the designed opposed flow velocity and oxygen concentration were applied. In the experiment, the opposed flow was increased subsequently until the flame was blow-off. The same Nikon D300 camera was used to record the flame-spread process. All normal-gravity experiments were repeated at least three times to reduce the random error.

## Results

**Figure 4:**
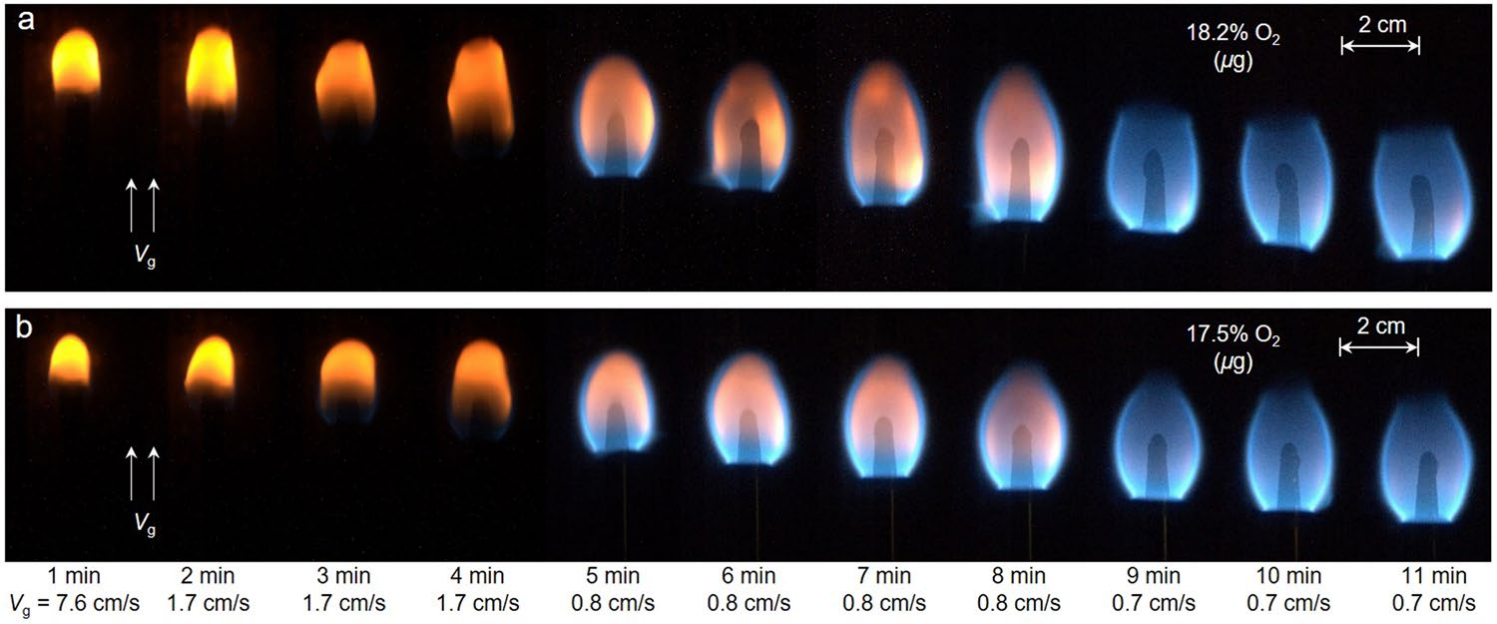
Photo sequence of microgravity flame spread under various opposed flow velocity (**a**) ${X}_{{{\rm{O}}}_{2}}$ = 18.2% (Sample B16), and (**b**) ${X}_{{{\rm{O}}}_{2}}$ = 17.5% (Sample B19).

Figure 4 shows a sequence of photos from an opposed flame spread in microgravity over a 0.64 mm diameter black cast PMMA rod at two oxygen concentrations (${X}_{{{\rm{O}}}_{2}}$) of 17.5% and 18.2%. For both tests, the opposed flow velocity (***V***_***g***_) was initially 7.6 cm/s (left) and was subsequently incrementally reduced to 0.7 cm/s (right). Each time step was at least 1 min that allowed the flame to reach quasi-steady state. The flame eventually extinguished when the flow velocity was reduced below 0.6 cm/s.

It is seen that in microgravity as the flow velocity is decreased, the flame color changes from yellow to blue, and the flame tip becomes open. Since the yellow color in a flame is primarily a result of soot radiation^27^, it may be inferred that the soot production and the flame temperature are significantly reduced as the flow velocity is decreased below 1 cm/s. Reducing the flow velocity further, the flame becomes longer, and its shape becomes more spherical. Compared to the flame in normal gravity (Fig. 1), the microgravity flame color is blue, indicating a lower soot concentration in the flame or potential soot standing away from hotter regions^28^. Moreover, the microgravity flame length is much smaller without a long tail, and the flame width is larger.

Multiple tests were conducted for each PMMA sample by keeping the airflow of constant oxygen concentration. From the videos of the different tests, the flame spread rate was measured by tracking the flame leading edge from high-resolution photos (about every 1 s) using an imaging tracking code. The complete tracking of the flame position in Fig. 4 as a function of time is provided in Fig. S1. The linear dependence of the flame position with time indicates a quasi-steady-state flame spread.

**Figure 5:**
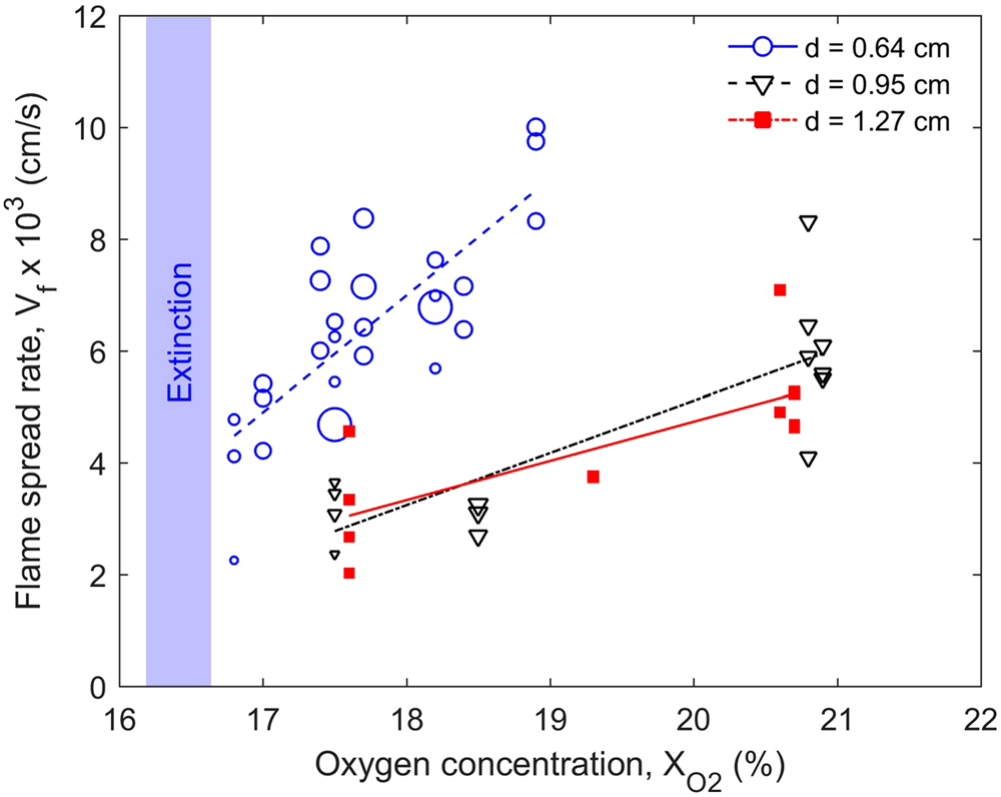
The measured microgravity flame-spread rate as a function of oxygen concentration and sample diameter where the size of symbol indicates the relative magnitude of the opposed flow velocity in the range of 0.4 and 7.6 cm/s.

The microgravity experimental procedure was decided to optimize the short astronaut time available to conduct the tests, the limited number of samples available, and the limited resources of oxygen. This resulted in data points that were not systematically taken, and that was difficult to process and present in an organized fashion. Also, it was difficult to determine how accurate were the values for oxygen concentration and flow velocity, because of potential changes in experimental conditions (cleanliness of the filters, settings in the flow and oxygen meters, etc). The complete set of the measured flame spread rate under various sample diameter, oxygen concentration, and opposed flow velocity is plotted in Fig. 5. The detailed information of tests and the processed flame spread rate for each test is summarized in Tables S1 (μg) and S2 (1 g).

To improve the analysis of the data, multivariate linear regression was undertaken, with oxygen concentration, opposed flow velocity, and sample diameter as independent variables, and flame spread rate as the dependent variable. This regression showed the statistical significance of the effects of each of the three independent variables against the dependent variable, and was used to help analyze the data^22^. This procedure was applied to the data of Fig. 5, which shows the flame spread rate increasing almost linearly with the oxygen concentration. The flame spread over thin 0.64-cm sample is clearly faster than thick samples because of the curvature effect in flame heat flux.

**Figure 6:**
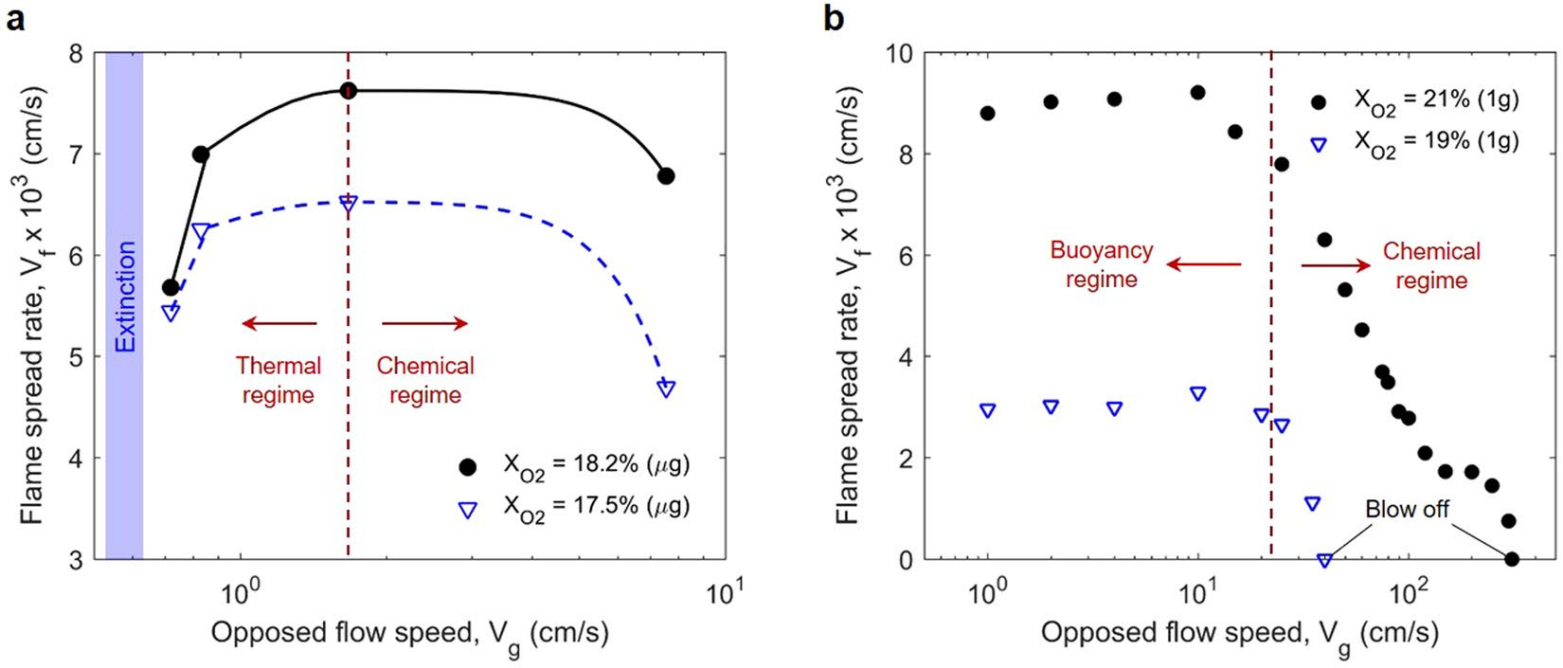
The measured flame-spread rate under various opposed flow velocity under (**a**) microgravity (μg) space station, ${X}_{{{\rm{O}}}_{2}}$ = 18.2% (Sample B16, 0.64 cm) and ${X}_{{{\rm{O}}}_{2}}$ = 17.5% (Sample B19, 0.64 cm), and (**b**) normal-gravity (1 g) Earth (1.27 cm) where flame cannot be sustained at ${X}_{{{\rm{O}}}_{2}}$ < 18.5%. Note that the scales of x axes in (**a**) and (**b**) are different.

Figure 6(a) shows the measured average flame-spread rate in microgravity (***V***_***f***_) versus the opposed flow velocity (***V***_***g***_) for the tests shown in Fig. 4. These two data sets were selected to present the nature of the results because they were tested under a larger range of the flow velocities and their measurements are more consistent.

The data show that for both oxygen concentrations, as the opposed flow velocity is increased, the flame spread rate first increases, and then decreases. No flame spread was observed at flow velocities less than 0.6 cm/s. The data in Fig. 6a are significant because of its uniqueness. More important, this trend of flame spread rate that first increases and then decreases with the flow velocity has only been predicted theoretically, but not been observed experimentally before^7,29,30^. Figure 6b shows the dependence of the flame-spread rate in normal gravity on the opposed flow velocity ranging from 0 to 500 cm/s. Note that the scales of x and y axes in Fig. 6a and b are different. It is seen that the flame spread behavior is different particularly at low flow velocities. In normal gravity, the upward buoyant flow generated by the flame itself dominates the low velocity forced flow, thus the flame spread rate is almost independent of the flow velocity when it is lower than the buoyant flow (~30 cm/s). Moreover, the low-flow extinction of the flame is not observed in normal gravity because the buoyant flow always presents.

**Figure 7:**
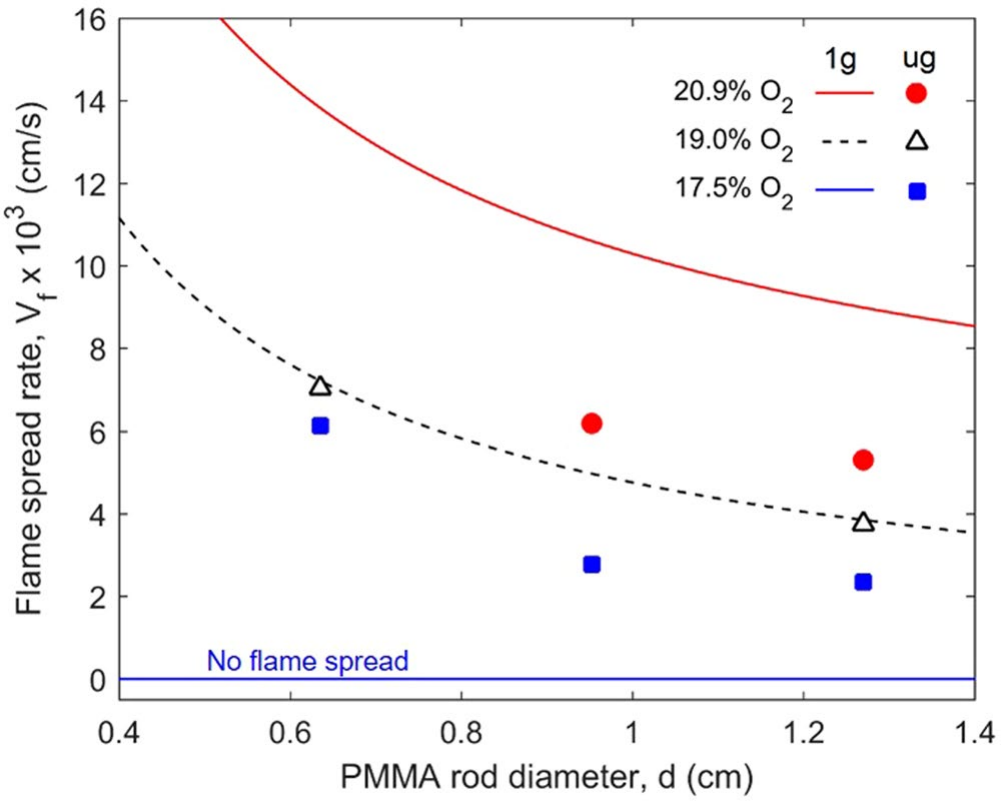
Variation of the opposed flame-spread rate with the rod diameter for black PMMA rods under microgravity (μg, symbols) and normal gravity (1 g, curves fitted from experimental data) where the opposed flow velocity is around 2 cm/s. Note that the uncertainty of oxygen concentration in microgravity is ±0.3%.

Figure 7 shows the measured dependence of the flame-spread rate with the rod diameter under normal gravity and microgravity, both at opposed flow velocities of around 2 cm/s. It is seen that the flame-spread rate increases as the rod diameter is decreased and that gravity level does not change such trend. There are two reasons (1) as the sample becomes thin^7^, the unburnt fuel ahead the flame becomes easy to preheat, and (2) as the rod diameter decreases, the convective boundary layer becomes thinner to increase the convective heating from the flame to the solid ahead of it (curvature effect, see the calculated convective heat transfer coefficient in Fig. S2)^15^. A similar effect of rod diameter on the normal gravity flame spread rate has been previously reported in the literature^10,15^.

Figure 7 also shows that for both gravity levels the flame spread is faster at higher oxygen concentration, as also previously observed in normal gravity^29^. As the oxygen concentration is increased, the flame temperature increases, as indicated by the brighter flame with higher soot concentrations. In turn, the convective and radiative heat transfers from the flame to the unburnt fuel ahead of the flame also increase. More importantly, it is found that flames in microgravity can spread at lower oxygen concentrations than in normal gravity. This is an important result because it indicates that the limiting oxygen concentration (LOC)^24^, below which flame spread cannot occur, is lower in microgravity than in normal gravity.

**Figure 8:**
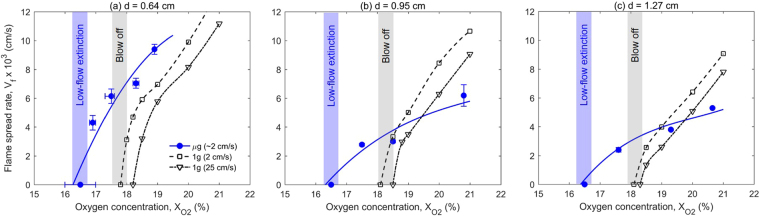
The flame-spread rates in microgravity (μg) and normal gravity (1 g) regarding the oxygen concentration for black cast PMMA rod diameter of (**a**) 0.64 cm, (**b**) 0.95 cm, and (**c**) 1.27 cm.

To further show this important result the dependence of the flame spread rate on oxygen concentration is presented in Fig. 8 for the different rod diameters in microgravity and normal gravity. It is seen in Fig. 8 that for all the diameters tested at ${{\rm{X}}}_{{{\rm{O}}}_{2}}$ = 21%, the flame spread is faster in normal gravity than in microgravity. However, below ${{\rm{X}}}_{{{\rm{O}}}_{2}}$ = 19% the flame spread becomes faster in microgravity than in normal gravity. More importantly, in microgravity, there is still flame spread at ${{\rm{X}}}_{{{\rm{O}}}_{2}}$ = 17%, while flame cannot exist in normal gravity for ${{\rm{X}}}_{{{\rm{O}}}_{2}}$ = 18% and below. Therefore, it can be concluded that at least for the present experimental conditions (low opposed flow) the LOC is lower (or the fire risk is higher) in microgravity than on Earth. This result may have significant implications for fire safety in spacecraft. For example, a larger amount of fire suppression agents such as CO_2_ or He gas or water mist, would be required to bring down the ambient oxygen level to extinguish the fire.

## Discussion

### Flame spread

Theoretically, the opposed flame spread can be analyzed as a continuous ignition process where the flame acts both as the source of solid fuel heating and pyrolysis, and the pilot for the ignition of the resulting pyrolysate-air mixture ahead of the flame^6,7^. Using phenomenological arguments^7^, it has been proposed that as the opposed gas flow velocity is increased from relatively low flow velocities to large flow velocities, the flame spread rate is controlled by two opposing mechanisms, one the heat transfer mechanisms from the flame to the solid ahead of the flame, the “Thermal Regime,” and the other the gas phase chemical kinetics ahead of the spreading flame, the “Chemical Regime^7,20,29^”. The thermal regime is characterized by a non-dimensional heat transfer number that is a ratio of the heat flux on the solid surface to the heat required to heat the solid to its pyrolysis temperature. The gas flow velocity appears in the former parameter and the flame spread velocity in the later. The chemical regime is characterized by the convective Damkohler number^27^,

1 $${\boldsymbol{Da}}=\frac{{{\boldsymbol{t}}}_{{\rm{chem}}}}{{{\boldsymbol{t}}}_{{\rm{flow}}}}\,$$

which is the ratio of the chemical time (***t***_chem_) to the flow time with a characteristic flow time (***t***_flow_) based on convection.

With relatively low flow velocities, increasing the opposed flow velocity moves the flame closer to the solid surface increasing the heat transfer from the flame to the solid ahead, counteracting the heat losses from the surface, and enhancing the solid pyrolysis. It also enhances the reaction rate and the heat released by the flame. This results in the flame spread rate increasing as the opposed flow velocity is increased^29–31^. This flame spread regime is often termed the “Thermal Regime”. On the other hand, at relatively low oxygen concentrations and/or when the opposed flow velocity is relatively large, increasing the opposed flow velocity slows down the gas phase chemical reaction by cooling. As a result, the flame becomes weaker, the flame spread becomes slower, and eventually the opposed flow blows off the flame. As the oxygen concentration decreases, the blow-off velocity decreases because both the reaction temperature and reaction rate decrease. Such flame spread behavior is often termed as the “Chemical Regime”. The interaction of these two regimes results in a flame spread rate that first increases with the opposed flow velocity and then decreases. The location of the turning point varies with the oxygen concentration, i.e., moving to higher flow velocities as the oxygen concentration increases. This flame spread behavior has been predicted by numerical simulations (see Fig. S3)^32,33^. However, at relatively low flow velocities and low oxygen concentrations the Thermal-Regime behavior had not been verified experimentally before for thick fuels until the present microgravity tests (Fig. 6a) because buoyancy prevents obtaining low flow velocities on Earth. Also, in microgravity not only the flame-spread rate, but the flame shape and color are also found to change significantly with the opposed flow if the flow velocity is low (***V***_***g***_ < 10 cm/s), as seen from Figs 1 and 4.

In normal gravity and at low flow velocities, there is almost no change in the flame shape, color and spread rate. In fact, as long as a flame can exist in normal gravity, there is a minimum buoyant flow (~30 cm/s) induced by flame, dominating over any opposed forced flow smaller than that. Therefore, at low flow velocities, flame characteristics and flame-spread rate become insensitive to the applied flow (see Figs 6b and S2)^10^. This buoyant flow also prevents the low-velocity extinction which only occurs in microgravity. Thus, the low-velocity regime should be renamed as the “Buoyancy Regime” that for the present experiments can be clearly observed at *V*_*g*_ < 25 cm/s. As the flow velocity is further increased, the Chemical Regime determines the flame behavior in normal gravity. However, the transition to the Chemical Regime occurs at higher forced flow velocity in normal gravity than in micro-gravity, due to the contribution of the buoyant-flow velocity to the forced-flow velocity (mixed forced and buoyant flow).

In a relatively high oxygen concentration (${{\rm{X}}}_{{{\rm{O}}}_{2}} > 19 \%$), the flame spread is faster in normal gravity than in microgravity (Fig. 8). It is because in normal gravity the strong buoyancy flow reduces the flame boundary layer thickness, and increases the heat flux from the flame. Comparatively, in microgravity and in low flow velocity, the flame standoff distance is larger (Figs 1 and 4) and the heat flux from flame is smaller. On the other hand, in a low oxygen concentration (${{\rm{X}}}_{{{\rm{O}}}_{2}} < 19 \%$), the flame spread rate is smaller in normal gravity than that in microgravity (Fig. 8), probably because the already weak flame becomes cooled by the strong buoyant flow. Then, the flame spread rate quickly decreases as the oxygen concentration decreases near its extinction limit. In other words, near the LOC, the slow chemistry also controls the flame spread (discussed more below).

### Extinction

For microgravity at very small opposed flow, the mechanism for flame extinction is different. Under these conditions, the flame is weak and is located away from the solid surface due to the thicker boundary layer. Also, oxygen starvation contributes to the flame moving outward seeking for oxygen. Consequently, changes in both transport and chemistry could contribute to the extinction. As the boundary layer thickens and the flame moves away from the surface, the heat transfer from the flame to the surface decreases, and heat losses primarily by radiation from the surface to the environment control the heat transfer number reducing its value. This causes a very slow flame spread and the flame extinction at a limiting low flow velocity. This very low flow velocity regime has been termed by some researchers as “radiation extinction”^20,22^, or “oxygen-transport limited flame spread region”^34^. The Damkohler number associated with these conditions is also affected. The transport of species to the flame would be a combination of convection and diffusion, and consequently the characteristic flow time would be a combination of diffusion and convection times. Also, the slow transport of oxygen to the reaction zone (flame) would also contributes to the presence of weak flame, and its extinction by heat losses to the surrounded gas if the reaction rate is too slow^18^. To further quantify the contributions of the slow transport of oxygen and radiative heat loss, more advanced diagnostic tool is desired in future microgravity experiment^28^. The microgravity flame extinction at high flow velocities (blow off) was not observed because of the limited number of tests and limited power of fan in ISS.

In normal gravity, reducing the oxygen concentration to 18%, a different mechanism of flame extinction may appear. In a low oxygen concentration, the flame-induced buoyant flow velocity (~30 cm/s) can exceed its blow-off limit and reduce the flame temperature below about 1130 K^35^, eventually, extinguish the flame. This extinction may be called as the self-induced buoyant blow-off, which has been also observed in the upward flame spread on thin solid fuels^36^. In contrast, in absence of buoyant flow, the flame spread in microgravity can be sustained at a lower oxygen concentration (${X}_{{O}_{2}}\le \mathrm{17} \%$, see Fig. 8), although only at low flow velocities.

## Concluding Remarks

The opportunity to conduct flame spread experiments in the ISS has provided data in micro-gravity low velocity flows that although limited in scope has significant information about the flammability of materials in spacecraft environments. The study has provided opposed flame spread data in microgravity that are rare and very valuable for the verification of theoretical models of flame spread over thick solid combustible materials. The data are rare because it is very difficult to conduct experiments in microgravity and for long enough time to measure flame spread rates over thick solids. These data are also valuable to verify theoretical models because at normal gravity it is not possible to test flame spread in flows with velocities lower than those induced by buoyancy (at least 30 cm/s).

Previous work has shown that the ignition time in microgravity for a solid combustible exposed to an external radiant flux in low-velocity oxidizing flows is shorter than in normal gravity^16^. This together with a faster flame spread rate as found for oxygen concentrations below 19% and a lower LOC implies that under certain environmental conditions combustible materials may be more flammable in a spacecraft than on Earth, i.e., a higher fire risk. Moreover, a lower LOC indicates that it may be more difficult to suppress the fire in a spacecraft than on Earth since a larger amount of fire suppression agents such as CO_2_ or He gas or water mist, would be required to bring down the ambient oxygen concentration to extinguish the fire. Thus, the results presented here may have significant implications for fire safety in spacecraft.

## Electronic supplementary material

Supplementary information
