# Supplementary material for: The Effect of Gravity on Flame Spread over PMMA Cylinders
Source: Sci Rep. 2018 Jan 9;8:120. doi: 10.1038/s41598-017-18398-4 (PMC5760683; doi:10.1038/s41598-017-18398-4)
Supplement: Supplementary file 1 — Supplementary information [file 41598_2017_18398_MOESM1_ESM.pdf]

# The Effect of Gravity on Flame Spread over PMMA Cylinders

**Shmuel Link[1], Xinyan Huang[1,*], Carlos Fernandez-Pello[1], Sandra Olson[2], and Paul Ferkul[2]**

[1]*University of California Berkeley Department of Mechanical Engineering, Berkeley, CA, USA*

[2] *NASA Glenn Research Center at Lewis Field, Cleveland, OH, USA.*

*xinyan.huang@berkeley.edu

## Supplemental Material

**Table S1:** The raw and processed microgravity data in International Space Station, where B and C means black and clear PMMA samples, respectively.

| $d$ (cm) | $X_{O_2}$ (%) | Sample No. | $V_g$ (cm/s) | $\Delta t$ (s) | $V_f \times 10^3$ (cm/s) | $d$ (cm) | $X_{O_2}$ (%) | Sample No. | $V_g$ (cm/s) | $\Delta t$ (s) | $V_f \times 10^3$ (mm/s) |
|---|---|---|---|---|---|---|---|---|---|---|---|
| 1.27 | 20.7 | B13a | 1.64 | 184 | 5.23 | 0.64 | 18.9 | B6 | 2.00 | 90 | 10.00 |
| | | | 1.54 | 232 | 5.27 | | | | 1.87 | 121 | 9.74 |
| | | | 1.43 | 239 | 4.63 | | | | 1.73 | 93 | 8.32 |
| | | | 1.38 | 238 | 4.69 | | 18.4 | B15 | 2.14 | 424 | 7.16 |
| | 20.6 | B13b | 1.64 | 159 | 7.09 | | | B14 | 1.98 | 384 | 6.38 |
| | | | 1.54 | 179 | 4.91 | | 18.2 | B16 | 7.55 | 72 | 6.78 |
| | 19.3 | B5 | 1.64 | 192 | 3.76 | | | | 1.67 | 139 | 7.62 |
| | | | 1.54 | 241 | 3.74 | | | | 0.83 | 198 | 6.99 |
| | 17.6 | B9 | 1.64 | 147 | 4.57 | | | | 0.72 | 170 | 5.68 |
| | | | 1.54 | 76 | 3.34 | | 17.7 | B1 | 4.00 | 794 | 7.15 |
| | | | 1.43 | 154 | 2.67 | | | | 2.15 | 23 | 5.91 |
| | | | 1.38 | 112 | 2.03 | | | C3a | 2.50 | 33 | 8.37 |
| 0.95 | 20.9 | C25 | 1.58 | 115 | 6.09 | | | | 2.00 | 30 | 6.42 |
| | | | 1.52 | 169 | 5.50 | | 17.5 | B19 | 7.55 | 67 | 4.69 |
| | | | 1.48 | 181 | 5.59 | | | | 1.67 | 128 | 6.52 |
| | 20.8 | B22 | 1.58 | 123 | 6.45 | | | | 0.83 | 189 | 6.25 |
| | | | 1.58 | 79 | 8.31 | | | | 0.72 | 200 | 5.44 |
| | | | 1.52 | 120 | 5.89 | | 17.4 | B2 | 2.50 | 24 | 7.25 |
| | | | 1.48 | 146 | 4.10 | | | | 2.00 | 28 | 7.87 |
| | 18.5 | B4 | 2.00 | 177 | 3.25 | | | C3b | 1.85 | 109 | 6.00 |
| | | | 1.87 | 131 | 3.10 | | 17.0 | B10 | 2.00 | 106 | 5.41 |
| | | | 1.73 | 145 | 2.69 | | | | 1.87 | 104 | 5.15 |
| | 17.5 | B12 | 1.01 | 121 | 3.08 | | | | 1.73 | 148 | 4.22 |
| | | | 0.82 | 134 | 3.45 | | 16.8 | P17 | 1.01 | 123 | 4.12 |
| | | | 0.60 | 129 | 3.65 | | | | 0.82 | 187 | 4.78 |
| | | | 0.48 | 158 | 2.37 | | | | 0.41 | 65 | 2.26 |

**Table S2:** The normal gravity experimental data of black PMMA rod samples on Earth where the reported flame spread rate is the average of at least 3 repeating tests and the flame spread over sample at stead state for at least 5 min or 4 cm.

| $d$ (cm) | $X_{O_2}$ (%) | $V_g$ (cm/s) | $V_f \times 10^3$ (cm/s) | ± (%) | $d$ (cm) | $X_{O_2}$ (%) | $V_g$ (cm/s) | $V_f \times 10^3$ (cm/s) | ± (%) |
|---|---|---|---|---|---|---|---|---|---|
| 1.27 | 20.9 | 25 | 7.81 | 0.2 | 0.95 | 20.9 | 25 | 9.06 | 4.3 |
| | | 2 | 9.07 | 2.5 | | | 2 | 10.65 | 0.3 |
| | 20.0 | 25 | 5.08 | 0.8 | | 20.0 | 25 | 6.29 | 0.6 |
| | | 2 | 6.41 | 0.2 | | | 2 | 8.45 | 0.4 |
| | 19.0 | 25 | 2.60 | 2.9 | | 19.0 | 25 | 3.50 | 1.0 |
| | | 2 | 3.99 | 5.1 | | | 2 | 5.01 | 2.5 |
| | 18.5 | 25 | 1.35 | 8.0 | | 18.8 | 2 | 2.97 | 2.0 |
| | | 2 | 2.56 | 1.5 | | 18.5 | 25 | - | - |
| | 18.3 | 25 | - | - | | | 2 | 3.33 | 0.6 |
| | 18.1 | 2 | - | - | | 18.1 | 2 | - | - |
| 0.64 | 20.9 | 25 | 11.17 | 1.3 | 0.32 | 20.9 | 25 | 18.40 | 1.7 |
| | | 2 | 13.63 | 0.8 | | | 2 | 23.57 | 2.2 |
| | 20.0 | 25 | 8.16 | 0.6 | | 20.0 | 25 | 14.50 | 0.8 |
| | | 2 | 9.90 | 1.2 | | | 2 | 19.00 | 1.3 |
| | 19.0 | 25 | 5.77 | 2.2 | | 19.0 | 25 | 10.00 | 1.2 |
| | | 2 | 6.96 | 1.1 | | | 2 | 14.01 | 0.8 |
| | 18.5 | 25 | 3.20 | 3.0 | | 18.5 | 25 | 7.50 | 0.8 |
| | 18.5 | 2 | 5.90 | 0.5 | | | 2 | 11.02 | 0.3 |
| | 18.2 | 25 | - | - | | 18.0 | 25 | 4.35 | 0.8 |
| | 18.0 | 2 | 4.69 | 1.0 | | | 2 | 8.00 | 1.1 |
| | 18.0 | 2 | 3.13 | 2.3 | | 17.7 | 25 | - | - |
| | 17.8 | 2 | - | - | | 17.5 | 2 | 3.88 | 0.5 |
| | 17.5 | 2 | - | - | | 17.0 | 2 | - | - |

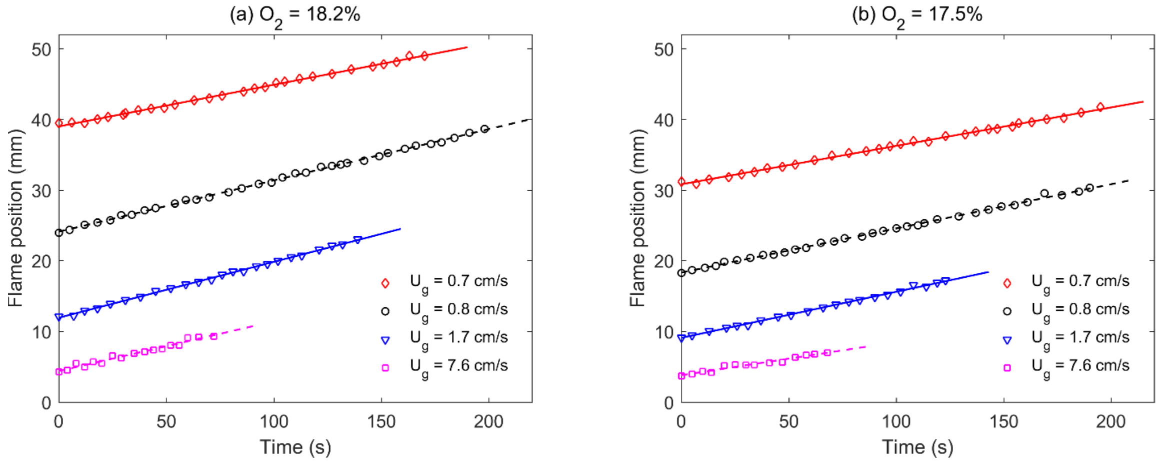


**Figure S1:** Tracking of the flame leading edge as a function of time through a black PMMA rod of 0.64 cm diameter under oxygen concentration ($X_{O_2}$) of (a) 18.2% (Sample B16) and (b) 17.5% (Sample B19).

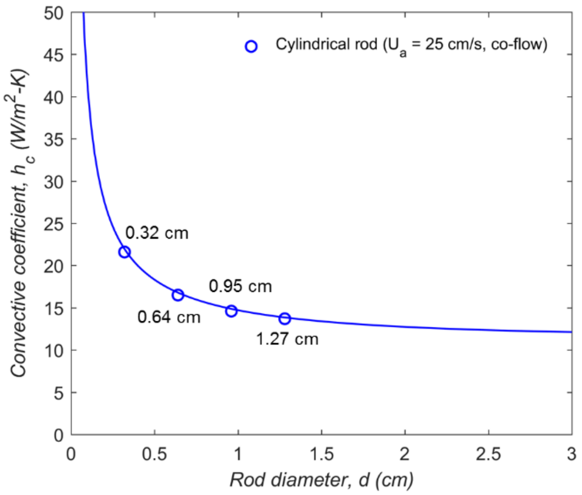


**Figure S2**. Convective heat transfer coefficient for the axial flow speed of 25 cm/s in normal gravity, calculated by the Direct Numerical Simulation (DNS).

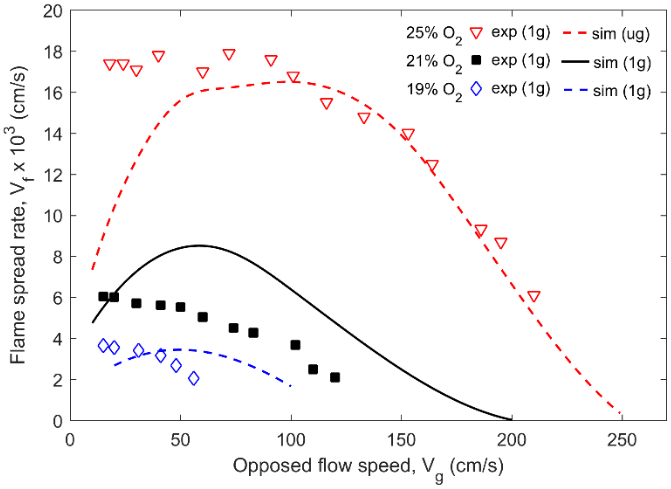


**Figure S3.** Comparison of modeled[1] (in microgravity) and experimental[2] (in normal gravity) flame spread rate in a flat PMMA plate as a function of opposed forced flow velocity at three oxygen concentrations. The model predicts there are both the increasing (thermal region) and the decreasing (chemical region) parts in microgravity. For the experiment on Earth, there is no thermal region as the flame spread rate is insensitive to the low flow velocity where the buoyancy flow controls.

## REFERENCES


1. Lautenberger, C., McAllister, S., Rich, D. & Fernandez-Pello, C. Modeling the Effect of Environmental Variables on Opposed-flow Flame Spread Rates with FDS. in *Proceedings of the International Conference on Fire Safety in Tall Buildings* 255–271 (2006).

2. Fernandez-Pello, A. C., Ray, S. R. & Glassman, I. Flame spread in an opposed forced flow: the effect of ambient oxygen concentration. *Symposium (International) on Combustion* **18,** 579–589 (1981).
